# Supplementary material for: Modeling glioblastoma heterogeneity as a dynamic network of cell states
Source: Mol Syst Biol. 2021 Sep 16;17(9):e10105. doi: 10.15252/msb.202010105 (PMC8444284; doi:10.15252/msb.202010105)
Supplement: Supplementary file 6 — Source Data for Figure 5 [file MSB-17-e10105-s004.zip › Figure5A_sourcedata/GSEA_3017/nosigdb_stateB.GseaPreranked.1622795021725/GAROFANO_IAVARONE_PPR_SUBTYPE.html]

Details for gene set GAROFANO\_IAVARONE\_PPR\_SUBTYPE[GSEA]

|  || Dataset | state43017 |
| Phenotype | NoPhenotypeAvailable |
| Upregulated in class | na\_pos |
| GeneSet | GAROFANO\_IAVARONE\_PPR\_SUBTYPE |
| Enrichment Score (ES) | 0.55699843 |
| Normalized Enrichment Score (NES) | 3.494044 |
| Nominal p-value | 0.0 |
| FDR q-value | 0.0 |
| FWER p-Value | 0.0 |
Table: GSEA Results Summary

  

Fig 1: Enrichment plot: GAROFANO\_IAVARONE\_PPR\_SUBTYPE      
 Profile of the Running ES Score & Positions of GeneSet Members on the Rank Ordered List

  

| PROBE | GENE SYMBOL | GENE\_TITLE | RANK IN GENE LIST | RANK METRIC SCORE | RUNNING ES | CORE ENRICHMENT || 1 | HIST1H4C |  |  | 0 | 1.373 | 0.0153 | Yes |
| 2 | HIST1H1B |  |  | 1 | 1.272 | 0.0294 | Yes |
| 3 | TOP2A |  |  | 3 | 1.079 | 0.0397 | Yes |
| 4 | RRM2 |  |  | 7 | 0.875 | 0.0442 | Yes |
| 5 | HIST2H2AC |  |  | 9 | 0.872 | 0.0521 | Yes |
| 6 | UBE2C |  |  | 11 | 0.802 | 0.0593 | Yes |
| 7 | TPX2 |  |  | 12 | 0.801 | 0.0682 | Yes |
| 8 | NUSAP1 |  |  | 13 | 0.801 | 0.0771 | Yes |
| 9 | HMGB2 |  |  | 14 | 0.786 | 0.0859 | Yes |
| 10 | NCAPG |  |  | 16 | 0.778 | 0.0928 | Yes |
| 11 | SPC25 |  |  | 17 | 0.763 | 0.1013 | Yes |
| 12 | PRC1 |  |  | 18 | 0.756 | 0.1097 | Yes |
| 13 | CDCA2 |  |  | 19 | 0.751 | 0.1180 | Yes |
| 14 | ESCO2 |  |  | 20 | 0.742 | 0.1263 | Yes |
| 15 | TACC3 |  |  | 21 | 0.741 | 0.1345 | Yes |
| 16 | GTSE1 |  |  | 22 | 0.732 | 0.1427 | Yes |
| 17 | HJURP |  |  | 23 | 0.731 | 0.1508 | Yes |
| 18 | KIF23 |  |  | 24 | 0.721 | 0.1588 | Yes |
| 19 | ASPM |  |  | 26 | 0.695 | 0.1648 | Yes |
| 20 | NDC80 |  |  | 27 | 0.695 | 0.1725 | Yes |
| 21 | ATAD2 |  |  | 28 | 0.685 | 0.1802 | Yes |
| 22 | KIF15 |  |  | 29 | 0.684 | 0.1878 | Yes |
| 23 | ARHGAP11A |  |  | 30 | 0.683 | 0.1954 | Yes |
| 24 | KPNA2 |  |  | 33 | 0.676 | 0.1994 | Yes |
| 25 | CENPF |  |  | 34 | 0.670 | 0.2068 | Yes |
| 26 | KIF4A |  |  | 37 | 0.662 | 0.2107 | Yes |
| 27 | RAD51AP1 |  |  | 40 | 0.657 | 0.2145 | Yes |
| 28 | SMC4 |  |  | 41 | 0.655 | 0.2218 | Yes |
| 29 | MELK |  |  | 42 | 0.655 | 0.2291 | Yes |
| 30 | CENPE |  |  | 43 | 0.654 | 0.2364 | Yes |
| 31 | NUF2 |  |  | 44 | 0.651 | 0.2436 | Yes |
| 32 | KIFC1 |  |  | 47 | 0.645 | 0.2473 | Yes |
| 33 | KIF18A |  |  | 48 | 0.639 | 0.2544 | Yes |
| 34 | NCAPG2 |  |  | 50 | 0.636 | 0.2597 | Yes |
| 35 | CENPA |  |  | 51 | 0.636 | 0.2668 | Yes |
| 36 | CDCA5 |  |  | 52 | 0.632 | 0.2738 | Yes |
| 37 | CKAP2 |  |  | 53 | 0.628 | 0.2808 | Yes |
| 38 | CCNA2 |  |  | 54 | 0.626 | 0.2878 | Yes |
| 39 | FANCI |  |  | 56 | 0.615 | 0.2929 | Yes |
| 40 | MYBL2 |  |  | 58 | 0.612 | 0.2979 | Yes |
| 41 | CKAP2L |  |  | 59 | 0.611 | 0.3047 | Yes |
| 42 | CENPK |  |  | 62 | 0.603 | 0.3079 | Yes |
| 43 | TYMS |  |  | 63 | 0.601 | 0.3146 | Yes |
| 44 | BUB1B |  |  | 64 | 0.596 | 0.3213 | Yes |
| 45 | KIF2C |  |  | 66 | 0.591 | 0.3261 | Yes |
| 46 | SMC2 |  |  | 68 | 0.586 | 0.3308 | Yes |
| 47 | CDK1 |  |  | 69 | 0.585 | 0.3374 | Yes |
| 48 | H2AFX |  |  | 71 | 0.576 | 0.3420 | Yes |
| 49 | CKS1B |  |  | 72 | 0.575 | 0.3484 | Yes |
| 50 | RACGAP1 |  |  | 75 | 0.572 | 0.3513 | Yes |
| 51 | FOXM1 |  |  | 76 | 0.570 | 0.3576 | Yes |
| 52 | MAD2L1 |  |  | 77 | 0.569 | 0.3639 | Yes |
| 53 | PLK1 |  |  | 78 | 0.567 | 0.3702 | Yes |
| 54 | DBF4 |  |  | 79 | 0.567 | 0.3765 | Yes |
| 55 | CDCA8 |  |  | 82 | 0.562 | 0.3793 | Yes |
| 56 | ECT2 |  |  | 84 | 0.558 | 0.3838 | Yes |
| 57 | KIF14 |  |  | 85 | 0.557 | 0.3900 | Yes |
| 58 | UBE2S |  |  | 87 | 0.555 | 0.3944 | Yes |
| 59 | AURKB |  |  | 89 | 0.548 | 0.3987 | Yes |
| 60 | PIF1 |  |  | 90 | 0.545 | 0.4048 | Yes |
| 61 | SPC24 |  |  | 91 | 0.544 | 0.4109 | Yes |
| 62 | CENPM |  |  | 92 | 0.544 | 0.4169 | Yes |
| 63 | NCAPH |  |  | 94 | 0.543 | 0.4212 | Yes |
| 64 | XRCC2 |  |  | 98 | 0.530 | 0.4218 | Yes |
| 65 | SKA3 |  |  | 99 | 0.525 | 0.4277 | Yes |
| 66 | RRM1 |  |  | 103 | 0.520 | 0.4282 | Yes |
| 67 | CCNF |  |  | 106 | 0.513 | 0.4304 | Yes |
| 68 | CEP135 |  |  | 109 | 0.513 | 0.4326 | Yes |
| 69 | UBE2T |  |  | 110 | 0.512 | 0.4383 | Yes |
| 70 | BUB1 |  |  | 113 | 0.508 | 0.4405 | Yes |
| 71 | BIRC5 |  |  | 116 | 0.504 | 0.4426 | Yes |
| 72 | CLSPN |  |  | 117 | 0.503 | 0.4482 | Yes |
| 73 | DTYMK |  |  | 118 | 0.503 | 0.4538 | Yes |
| 74 | CEP152 |  |  | 119 | 0.502 | 0.4594 | Yes |
| 75 | ASF1B |  |  | 120 | 0.501 | 0.4650 | Yes |
| 76 | KIF18B |  |  | 123 | 0.494 | 0.4670 | Yes |
| 77 | DUT |  |  | 124 | 0.494 | 0.4725 | Yes |
| 78 | E2F8 |  |  | 125 | 0.493 | 0.4779 | Yes |
| 79 | TMPO |  |  | 127 | 0.492 | 0.4817 | Yes |
| 80 | LMNB2 |  |  | 129 | 0.488 | 0.4853 | Yes |
| 81 | SKA1 |  |  | 131 | 0.487 | 0.4890 | Yes |
| 82 | GMNN |  |  | 135 | 0.484 | 0.4891 | Yes |
| 83 | FANCB |  |  | 138 | 0.478 | 0.4910 | Yes |
| 84 | INCENP |  |  | 140 | 0.476 | 0.4945 | Yes |
| 85 | FANCD2 |  |  | 149 | 0.461 | 0.4857 | Yes |
| 86 | KIF22 |  |  | 152 | 0.459 | 0.4873 | Yes |
| 87 | NCAPD3 |  |  | 153 | 0.458 | 0.4924 | Yes |
| 88 | CDC45 |  |  | 154 | 0.456 | 0.4974 | Yes |
| 89 | SPAG5 |  |  | 156 | 0.455 | 0.5008 | Yes |
| 90 | CEP78 |  |  | 161 | 0.451 | 0.4988 | Yes |
| 91 | POLQ |  |  | 162 | 0.450 | 0.5038 | Yes |
| 92 | TK1 |  |  | 167 | 0.444 | 0.5017 | Yes |
| 93 | BRIP1 |  |  | 168 | 0.443 | 0.5067 | Yes |
| 94 | ATAD5 |  |  | 170 | 0.442 | 0.5098 | Yes |
| 95 | BRCA1 |  |  | 171 | 0.440 | 0.5147 | Yes |
| 96 | BRCA2 |  |  | 174 | 0.436 | 0.5161 | Yes |
| 97 | DEPDC1B |  |  | 177 | 0.436 | 0.5174 | Yes |
| 98 | MKI67 |  |  | 179 | 0.436 | 0.5205 | Yes |
| 99 | FEN1 |  |  | 180 | 0.435 | 0.5254 | Yes |
| 100 | BARD1 |  |  | 189 | 0.427 | 0.5161 | Yes |
| 101 | CKAP5 |  |  | 192 | 0.426 | 0.5174 | Yes |
| 102 | MND1 |  |  | 193 | 0.424 | 0.5221 | Yes |
| 103 | MCM4 |  |  | 199 | 0.418 | 0.5180 | Yes |
| 104 | WDR62 |  |  | 200 | 0.415 | 0.5226 | Yes |
| 105 | DSN1 |  |  | 202 | 0.413 | 0.5254 | Yes |
| 106 | ZWINT |  |  | 212 | 0.407 | 0.5142 | Yes |
| 107 | LIG1 |  |  | 214 | 0.407 | 0.5170 | Yes |
| 108 | H2AFZ |  |  | 216 | 0.405 | 0.5198 | Yes |
| 109 | MNS1 |  |  | 219 | 0.401 | 0.5207 | Yes |
| 110 | CENPO |  |  | 223 | 0.398 | 0.5199 | Yes |
| 111 | FANCA |  |  | 227 | 0.395 | 0.5191 | Yes |
| 112 | TIMELESS |  |  | 228 | 0.395 | 0.5235 | Yes |
| 113 | STIL |  |  | 231 | 0.393 | 0.5243 | Yes |
| 114 | PKMYT1 |  |  | 233 | 0.390 | 0.5269 | Yes |
| 115 | POC1A |  |  | 234 | 0.390 | 0.5313 | Yes |
| 116 | EZH2 |  |  | 236 | 0.388 | 0.5338 | Yes |
| 117 | CDC6 |  |  | 239 | 0.388 | 0.5346 | Yes |
| 118 | TRIP13 |  |  | 241 | 0.387 | 0.5372 | Yes |
| 119 | PCNA |  |  | 242 | 0.386 | 0.5415 | Yes |
| 120 | HMGN2 |  |  | 243 | 0.386 | 0.5458 | Yes |
| 121 | POLA1 |  |  | 247 | 0.383 | 0.5448 | Yes |
| 122 | RTKN2 |  |  | 248 | 0.379 | 0.5490 | Yes |
| 123 | CCNB2 |  |  | 250 | 0.379 | 0.5515 | Yes |
| 124 | POLD3 |  |  | 252 | 0.377 | 0.5539 | Yes |
| 125 | PLK4 |  |  | 255 | 0.374 | 0.5546 | Yes |
| 126 | C18orf54 |  |  | 259 | 0.371 | 0.5535 | Yes |
| 127 | CENPJ |  |  | 261 | 0.370 | 0.5558 | Yes |
| 128 | DPF1 |  |  | 264 | 0.367 | 0.5564 | Yes |
| 129 | CENPW |  |  | 267 | 0.366 | 0.5570 | Yes |
| 130 | SMC1A |  |  | 274 | 0.361 | 0.5505 | No |
| 131 | CHAF1A |  |  | 276 | 0.359 | 0.5528 | No |
| 132 | ANP32E |  |  | 280 | 0.358 | 0.5515 | No |
| 133 | GINS1 |  |  | 283 | 0.357 | 0.5520 | No |
| 134 | ZWILCH |  |  | 292 | 0.353 | 0.5419 | No |
| 135 | HAUS8 |  |  | 293 | 0.353 | 0.5459 | No |
| 136 | CENPP |  |  | 299 | 0.348 | 0.5410 | No |
| 137 | NEIL3 |  |  | 304 | 0.345 | 0.5378 | No |
| 138 | DTL |  |  | 305 | 0.345 | 0.5417 | No |
| 139 | CDC25C |  |  | 307 | 0.343 | 0.5437 | No |
| 140 | CENPN |  |  | 309 | 0.343 | 0.5458 | No |
| 141 | FAM111B |  |  | 314 | 0.342 | 0.5426 | No |
| 142 | OIP5 |  |  | 315 | 0.341 | 0.5464 | No |
| 143 | RFC3 |  |  | 322 | 0.338 | 0.5397 | No |
| 144 | RFC2 |  |  | 331 | 0.333 | 0.5294 | No |
| 145 | HIRIP3 |  |  | 336 | 0.331 | 0.5261 | No |
| 146 | HAUS3 |  |  | 351 | 0.325 | 0.5052 | No |
| 147 | LMNB1 |  |  | 363 | 0.321 | 0.4896 | No |
| 148 | PNRC2 |  |  | 364 | 0.321 | 0.4931 | No |
| 149 | POLA2 |  |  | 380 | 0.314 | 0.4704 | No |
| 150 | BLM |  |  | 408 | 0.305 | 0.4266 | No |
| 151 | LIN54 |  |  | 411 | 0.303 | 0.4265 | No |
| 152 | DSCC1 |  |  | 422 | 0.299 | 0.4123 | No |
| 153 | CDKN1B |  |  | 427 | 0.298 | 0.4086 | No |
| 154 | SASS6 |  |  | 432 | 0.296 | 0.4049 | No |
| 155 | RECQL4 |  |  | 443 | 0.294 | 0.3907 | No |
| 156 | CCDC150 |  |  | 449 | 0.293 | 0.3853 | No |
| 157 | CBX5 |  |  | 457 | 0.291 | 0.3763 | No |
| 158 | SAE1 |  |  | 463 | 0.289 | 0.3707 | No |
| 159 | RFWD3 |  |  | 464 | 0.289 | 0.3739 | No |
| 160 | ING2 |  |  | 466 | 0.289 | 0.3754 | No |
| 161 | ZNF107 |  |  | 472 | 0.287 | 0.3699 | No |
| 162 | CHEK1 |  |  | 477 | 0.286 | 0.3660 | No |
| 163 | RNASEH2A |  |  | 478 | 0.285 | 0.3692 | No |
| 164 | NUCKS1 |  |  | 483 | 0.285 | 0.3654 | No |
| 165 | CDT1 |  |  | 486 | 0.284 | 0.3651 | No |
| 166 | CTDSPL2 |  |  | 495 | 0.282 | 0.3542 | No |
| 167 | DBF4B |  |  | 498 | 0.281 | 0.3538 | No |
| 168 | CENPH |  |  | 501 | 0.281 | 0.3535 | No |
| 169 | TNFAIP8L1 |  |  | 510 | 0.279 | 0.3426 | No |
| 170 | DNMT1 |  |  | 523 | 0.277 | 0.3247 | No |
| 171 | FIGNL1 |  |  | 542 | 0.273 | 0.2963 | No |
| 172 | SAP30 |  |  | 547 | 0.273 | 0.2923 | No |
| 173 | NIF3L1 |  |  | 561 | 0.270 | 0.2726 | No |
| 174 | CDK2 |  |  | 565 | 0.270 | 0.2703 | No |
| 175 | DDX11 |  |  | 568 | 0.269 | 0.2698 | No |
| 176 | NUP205 |  |  | 571 | 0.269 | 0.2693 | No |
| 177 | ZNF519 |  |  | 618 | 0.260 | 0.1918 | No |
| 178 | ICA1L |  |  | 621 | 0.259 | 0.1912 | No |
| 179 | CENPI |  |  | 630 | 0.258 | 0.1801 | No |
| 180 | ZNF85 |  |  | 635 | 0.258 | 0.1760 | No |
| 181 | EXOSC8 |  |  | 647 | 0.256 | 0.1596 | No |
| 182 | FAM76B |  |  | 653 | 0.255 | 0.1537 | No |
| 183 | PXMP2 |  |  | 663 | 0.253 | 0.1408 | No |
| 184 | RAD51 |  |  | 670 | 0.252 | 0.1331 | No |
| 185 | SPATA5 |  |  | 675 | 0.252 | 0.1289 | No |
| 186 | TUBA1A |  |  | 686 | -0.263 | 0.1143 | No |
| 187 | PCDH10 |  |  | 713 | -0.305 | 0.0723 | No |
| 188 | SOX11 |  |  | 754 | -0.576 | 0.0087 | No |
Table: GSEA details [plain text format]

  

Fig 2: GAROFANO\_IAVARONE\_PPR\_SUBTYPE: Random ES distribution      
 Gene set null distribution of ES for **GAROFANO\_IAVARONE\_PPR\_SUBTYPE**
